# Thermic effect of a meal and appetite in adults: an individual participant data meta-analysis of meal-test trials

**DOI:** 10.3402/fnr.v57i0.19676

**Published:** 2013-12-23

**Authors:** Anne-Marie Ravn, Nikolaj Ture Gregersen, Robin Christensen, Lone Graasbøl Rasmussen, Ole Hels, Anita Belza, Anne Raben, Thomas Meinert Larsen, Søren Toubro, Arne Astrup

**Affiliations:** 1 Faculty of Science, Department of Nutrition, Exercise and Sports, University of Copenhagen, Frederiksberg C, Denmark; 2 The Parker Institute: Musculoskeletal Statistics Unit, Copenhagen University Hospital at Bispebjerg and Frederiksberg, Copenhagen, Denmark; 3 StatistiConsult, Ølstykke, Denmark; 4 Reduce APS – Research Clinic of Nutrition, Roskilde, Denmark

**Keywords:** thermic effect of a meal, diet-induced thermogenesis, appetite, energy expenditure, satiety, Composite Appetite Score

## Abstract

**Background:**

Thermic effect of a meal (TEF) has previously been suggested to influence appetite.

**Objective:**

The aim of this study was to assess whether there is an association between appetite and TEF. Second, to examine whether protein intake is associated with TEF or appetite.

**Design:**

Individual participant data (IPD) meta-analysis on studies were performed at the Department of Nutrition, Exercise and Sports, University of Copenhagen, Denmark. Five randomized meal-test studies, with 111 participants, were included. The included studies measured energy expenditure (EE) in respiration chambers and pre- and postprandial appetite sensations using Visual Analog Scales (VAS). The primary meta-analysis was based on a generic-inverse variance random-effects model, pooling individual study Spearman's correlation coefficients, resulting in a combined *r*-value with 95% confidence interval (95% CI). The *I* ^2^ value quantifies the proportion (%) of the variation in point estimates due to among-study differences.

**Results:**

The IPD meta-analysis found no association between satiety and TEF expressed as the incremental area under the curve (TEF_iAUC_) (*r*=0.06 [95% CI −0.16 to 0.28], *P*=0.58; *I* ^2^=15.8%). Similarly, Composite Appetite Score (CAS) was not associated with TEF_iAUC_ (*r*=0.08 [95% CI −0.12 to 0.28], *P=*0.45; *I* ^2^=0%). Posthoc analyses showed no association between satiety or CAS and TEF expressed as a percentage of energy intake (EI) (*P*>0.49) or TEF expressed as a percentage of baseline EE (*P*>0.17). When adjusting for covariates, TEF_iAUC_ was associated with protein intake (*P*=0.0085).

**Conclusions:**

This IPD meta-analysis found no evidence supporting an association between satiety or CAS and TEF at protein intakes ∼15 E% (range 11–30 E%).

Human appetite involves repeated increases and decreases in the desire to eat and is controlled by psychological, physiological, and biochemical mechanisms (1, 2). Thermic effect of a meal (TEF) is the increase in energy expenditure (EE) engendered by the energy used in the postprandial period in the process of absorbing, metabolizing, and storing ingested nutrients (3, 4).

TEF has previously been suggested as one of the mechanisms that influences appetite sensations, including satiety (5, 6). Strominger and Brobeck (5) hypothesized that increased body temperature, caused by both environmental temperature and the extra heat released during the digestion of food (i.e. TEF), could result in a reduced food intake. Later, Westerterp-Plantenga et al. (6) suggested that the basis of the association between TEF and satiety might be that the increased EE at rest after food intake causes increased oxygen consumption and body temperature. This could then give rise to the body feeling deprived of oxygen and be translated into a sensation of satiety (6). The same relationship between oxygen deprivation and appetite has previously been observed in mountain climbers at high altitudes, although the causal association has not been thoroughly investigated (7, 8). Bearing the above in mind, it is of interest to consider the effect of diet, such as particular macronutrients, on TEF and consequently on appetite regulation.

Studies on appetite and thermogenesis have shown protein to be superior to other macronutrients in promoting satiety (2, 7). In addition, protein causes higher TEF (20–30% of the energy content of ingested protein) compared to carbohydrate (5–10%) and fat (0–3%) (9). This is due to the large amount of adenosine triphosphate (ATP) used in the postprandial period in the process of metabolizing and storing protein (9). As protein is the most satiating and thermogenic nutrient, the association between TEF and appetite suggested by Strominger and Brobeck in 1953 could be plausible (5).

Even a small positive association between TEF and satiety could have clinical implications during weight loss. We therefore undertook an individual participant data (IPD) meta-analysis on studies previously conducted at our department, to investigate whether an association between the perception of appetite and TEF exists, and whether protein intake is an influential factor.

## Present investigation

### Methods

#### Eligibility criteria

Studies conducted at the Department of Nutrition, Exercise and Sports, Faculty of Science, University of Copenhagen, Denmark, from 1992 to 2006 were available for inclusion in this study. Studies were included if they contained: 1) data on at least 24-h EE measurements in whole-body respiration chambers; 2) baseline measurements and a minimum of three measurements of appetite sensations after a dinner meal using visual analog scales (VAS); and 3) detailed descriptions of energy intake (EI). To obtain individual energy balance during the chamber stay, EI provided was based on equations estimating EE. In studies where active components (e.g. medication, enriched foods, etc.) were tested, only the control measurements done without these active components were included in this IPD meta-analysis. If more than one measurement was carried out on the same participant, and if both measurements matched the criteria above, only the last measurement was included. Finally, all included participants had to be healthy, non-smoking, and non-elite athletes (less than 10 h of exercise a week).

#### Respiratory measurements

EE was measured by indirect whole-body calorimetry in a 14.7 m^3^ open-circuit respiration chamber at the Faculty of Science (University of Copenhagen, Denmark). The design of the chambers has been described in detail elsewhere (10). In the included studies, oxygen and carbon dioxide exchange including urinary nitrogen measurements were used to calculate EE. Spontaneous physical activity (SPA) in the respiration chambers was assessed using two microwave radar devices. The within-subject variation for 24-h EE measured in the chambers is 2.3% and the within-subject variation for 1-h measurements of basal EE is 5% (10). Resting metabolic rate (RMR) was measured in a resting period of 30 min just before the dinner meal was served.

#### Visual analog scales

VAS were used for measuring the subjective appetite sensations of satiety, hunger, fullness, and prospective food intake. The scale consists of a horizontal line (100 mm in length) with the most positive and most negative sensations at opposite ends of the line. Participants mark the line at a point corresponding to their perceived appetite at a given time. For satiety, the question was ‘How satisfied do you feel?’, and the text anchors were ‘I am completely empty’ and ‘I cannot eat another bite’. For fullness, hunger and prospective food intake the questions were ‘How full do you feel?’ (text anchors: ‘Not at all full’ and ‘Totally full’), ‘How hungry do you feel?’ (text anchors: ‘I am not hungry at all’ and ‘I have never been hungrier’) and ‘How much do you think you could eat?’ (text anchors: ‘Nothing at all’ and ‘A lot’), respectively.

The reproducibility and validity of VAS have previously been examined by Flint et al. (11), who concluded that VAS provide a reliable tool for a quantitative measurement of subjective sensations such as appetite in single meal-test trials. Finally, Composite Appetite Score (CAS) was included in the study. This measure reflects the four VAS questions and was included in the study as a summary measure of appetite. CAS was calculated using the formula inspired by Anderson et al. (12): CAS=((100 − satiety)+(100 − fullness) + hunger + prospective food intake)/4.

#### Data

Data were acquired by contacting the principal investigators, searching old records, and contacting the research department where the studies were conducted. The data of all of the included participants were checked for duplicates and were checked twice (by a second reviewer) before being included in the IPD meta-analysis. Data were extracted from previously collected data and were treated as confidential.

Data on appetite (VAS scores on satiety and CAS [including satiety, fullness, hunger, and prospective food intake]) just before ingestion of the dinner meal and until 180 min after the meal were extracted from each of the included studies. Similarly, data on EE measurements 30 min before the dinner and until 180 min postprandially were also extracted. Furthermore, data on sex, age, height, body weight, EI, fat-free mass, fat mass, body fat percentage, and tea/coffee ingested after dinner (yes/no) were also collected. VAS was calculated as the incremental area under or over the curve (iAUC and iAOC, respectively) using VAS measurements filled out immediately before the dinner as baseline. The examined appetite measures were Satiety_iAUC_ and CAS.

TEF was calculated in three different ways. The primary TEF measure was calculated as the incremental area under the curve for resting EE after the dinner meal with RMR used as the baseline measure (TEF_iAUC_) and expressed in kJ/3 h. For the purpose of sensitivity analysis, we also scrutinized other ways to interpret TEF: (1) TEF as the increase in EE above baseline expressed as a percentage of EI in dinner meal (TEF%_EI_); and (2) TEF as the increase in EE above baseline expressed as a percentage of baseline (TEF%) as secondary TEF measures. These TEF measures were included in posthoc analyses of satiety and CAS. We preferred the dinner meal as this normally included 30–40% of the total EI during the stay.

#### Quality of included studies

All included studies were randomized controlled trials with either parallel (13–16) or crossover (17) design. Four of the studies used either a single- (15, 17) or double-blinded design (13, 14). Two of the studies were placebo-controlled trials testing potential drug candidates (13, 14). The remaining three studies all tested different diets against each other (15–17).

#### Statistical analysis

The raw data included from each study were analyzed separately to acquire summary statistics in the form of Spearman's *r*-coefficient and the corresponding standard error (SE), reflecting the level of statistical significance in the individual tests for association in the different eligible trials. The SE for the non-parametric Spearman's *r*-coefficient (SE[*r*]) was estimated from the (two-sided) *P*-value converted into a standard normal score (*Z*), which enabled an estimate of SE[*r*] using Wald-test methodology (*Z*[*r*]= *r*/SE[*r*]). All results are reported with 95% confidence intervals (95% CI), that were calculated as the estimate for coefficient *r*±1.96*SE. For the purpose of sensitivity analysis, these Wald-test-converted SEs were re-confirmed when estimated using the approach proposed by Thompson et al. (18) using the Fisher-transformed correlation (and SE) derived from the reported *P*-value and the sample size.

The summary analyses were computed using homogeneity statistics to evaluate the agreement of the individual trial results with a fixed-effect meta-analytic summary (19). However, for the overall inference we used standard random-effects meta-analysis (20) as a default option; the fixed-effect analysis would apply as a sensitivity analysis in the case of inconsistency. We estimated inconsistency by calculating the *I* ^2^ statistic (21), which describes the percentage of total variation across trials that is attributable to heterogeneity rather than to chance (22).

We performed a number of pre-specified sensitivity analyses based on a statistically more advanced hierarchical model. At level one in the hierarchical model, participants were compared with others from the same study (i.e. trial numbers were applied as clusters), enabling the entire dataset to be analyzed as if it originated from a single study (23). A random coefficient model was applied to assess the five different possible linear associations simultaneously (24). Random coefficient models emerge as natural mixed-model extensions of simple linear regression models in a hierarchical (nested) data setup. As we treated each study as a random sample, it was natural to incorporate this in the model by assuming the subject effects (trial intercepts and slopes) to be random (25). These models enabled inclusion of potentially confounding factors included in Table 1; factors and covariates that could influence the overall summary association across included studies (see Supplementary file). These hierarchical models derived from maximum likelihood estimates were performed using the SAS software (version 9.2).

**Table 1:** Summary of study characteristics of all participants

Variable	Raben et al. (15)	Hansen et al. (13)	Mikkelsen et al. (17)	Larsen et al. (14)	Rasmussen et al. (16)	Total
Publication year	2002	1999	2000	2002	2007	–
*N*	19	32	12	10	38	111
Males, no. (%)	3 (16%)	7 (22%)	12 (100%)	10 (100%)	16 (42%)	48 (43%)
Age, years	35.4±10.7 (20.0; 50.0)	38.5±9.05 (20.0; 54.0)	25.6±3.2 (21.0; 31.0)	36.1±7.6 (25.0; 47.0)	27.1±5.2 (18.0; 36.0)	32.3±9.4 (18.0; 54.0)
Caffeine (yes/no^1^)	Yes	No	No	Yes	No	–
SPA (%/h^2^)	7.4±3.6 (3.7; 19.3)	7.9±3.9 (2.9; 21.3)	8.0±1.7 ( 6.1; 11.2)	8.3±2.7 (4.7; 12.9)	6.3±2.0 (2.7; 10.8)	7.3±3.1 (2.7; 21.3)
BMI (kg/m^2^)	28.2±2.4 (24.3; 32.6)	33.8±2.7 (30.4; 40.0)	29.2±1.8 (26.8; 31.9)	31.3±2.1 (28.3; 34.3)	28.2±2.4 (22.5; 33.4)	30.2±3.4 (22.5; 40.0)
EI dinner (MJ^3^)	3.38±0.40 (2.81; 4.39)	4.26±0.48 (3.30; 5.18)	2.95±1.07 (1.07; 4.23)	5.91±0.39 (5.44; 6.43)	5.31±0.72 (4.23; 7.05)	4.48±1.14 (1.07; 7.05)
EI protein (MJ^4^)	0.48±0.06 (0.40; 0.62)	0.65±0.07 (0.51; 0.79)	0.68±0.35 (0.12; 1.24)	0.78±0.05 (0.71; 0.85)	0.80±0.11 (0.63; 1.05)	0.69±0.18 (0.20; 1.24)
Satiety iAUC^5^	7102.1±3212.3 (0; 11010.0)	5262.4±3171.7 (0; 11370.0)	6506.3±1675.3 (2535.0; 8535.0)	7911.0±2541.0 (3090.0; 11670.0)	8698.5±3092.3^9^ (170.0; 15015.0)	7097.9±3237.7^11^ (0; 15015.0)
CAS iAOC^6^	7057.9±2538.4 (3037.5; 10635.0)	5431.2±2952.5 (511.9; 10755.0)	6699.9±2294.4 (2267.9; 10338.8)	7101.7±2340.3^8^ (3052.5; 11580.0)	8439.5±3074.5^10^ (1372.8; 14340.0)	6986.9±3012.3^12^ (511.9; 14340.0)
TEF iAUC^7^	131.5±93.1 (1.0; 318.0)	168.0±91.1 (0; 482.0)	188.4±128.8 (46.0; 402.0)	168.2±124.4 (20.0; 398.0)	172.7±92.2 (20.0; 498.0)	165.6±99.1 (0; 498.0)

Values are mean±SD (min; max) unless otherwise stated.

1 One cup of tea/coffee containing caffeine was served during the measurement of diet-induced thermogenesis.

2 Spontaneous physical activity during the first 3 h after the dinner meal in percent per hour.

3 Energy intake from dinner meal in MJ.

4 Energy intake from protein in dinner meal in MJ.

5 The incremental area under the curve for satiety measured by visual analog scales.

6 Incremental area over the curve for the summary measure Composite Appetite Score. CAS=(satiety + fullness + hunger + prospective food intake)/4.

7 Diet-induced thermogenesis in kJ/3 h (incremental area under the curve for postprandial energy expenditure).

8 *n*=9 participants.

9 *n*=36 participants.

10 *n*=35 participants.

11 *n*=109 participants.

12 *n*=107 participants.

## Results

### Inclusion of studies

**Fig. 1:**
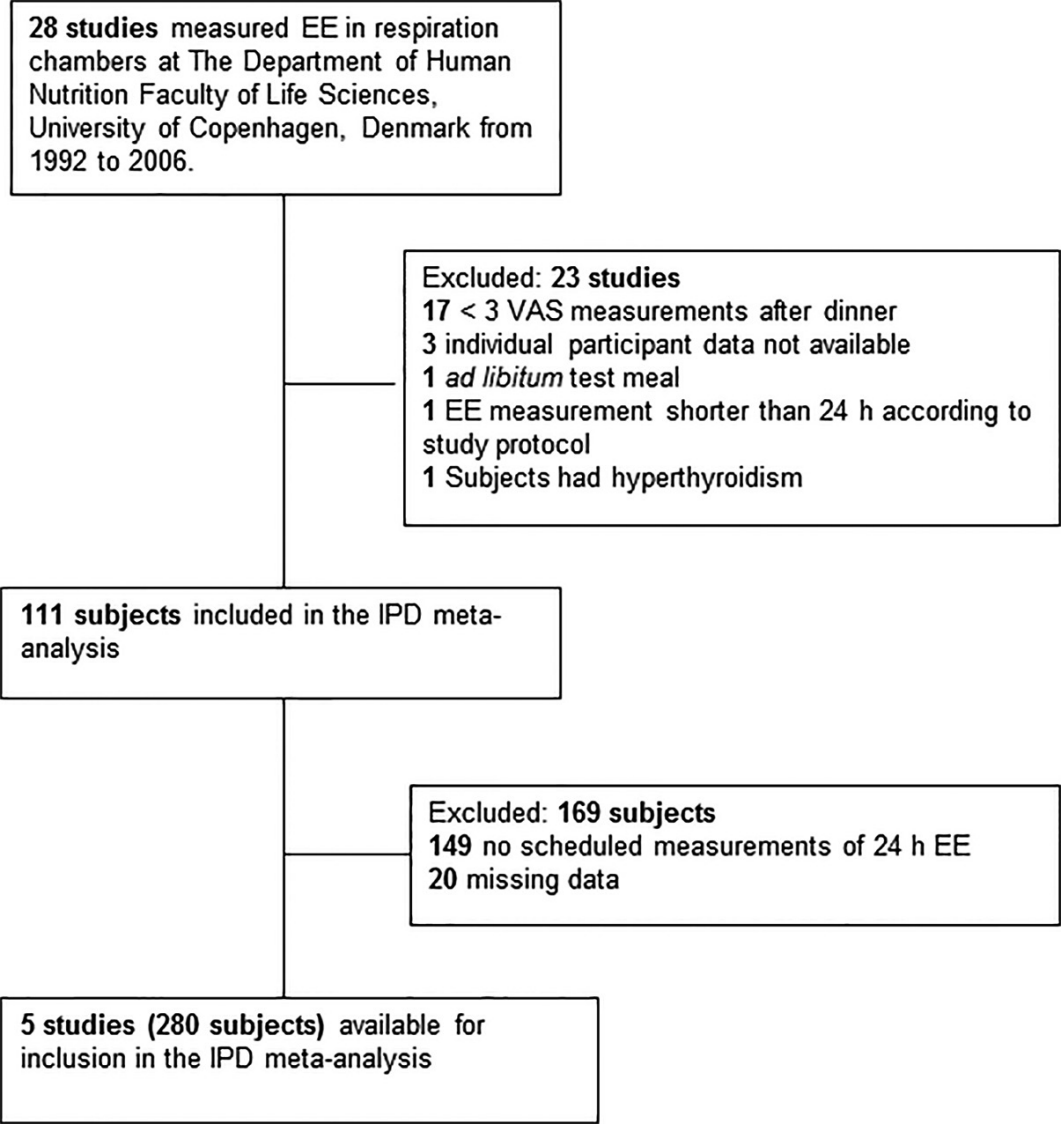
Flowchart of the selection and inclusion process.

A flowchart of the selection and inclusion process is given in Fig. 1. Twenty-eight studies included measurements from respiration chambers as a part of the investigations conducted at Department of Nutrition, Exercise and Sports in the period from 1992 to 2006 and therefore fulfilled these two inclusion criteria. However, 20 of these studies were excluded due to the following factors: less than three VAS measurements after the dinner meal (10, 26–41), *ad libitum* dinner meal (42), EE measurements shorter than 24 h (43), or the participants had hyperthyroidism (44). Furthermore, three studies were excluded because it was not possible to locate data (45). Thus, five studies were found eligible for inclusion in the meta-analysis. These five studies (13–17) included a total of 280 participants. In two of the five included studies, measurements in respiration chambers were carried out only on a subgroup of the included participants, thus 149 of the 280 participants were not measured in the respiration chambers and data on these participants was excluded. Furthermore, 20 participants were excluded due to missing data on EI, VAS scores, and body mass index (BMI). As a result, 111 healthy participants were included in this IPD meta-analysis. Study characteristics for each of the five included studies are listed in Table 1. Forty-eight of the 111 (43%) included participants were men. The mean (±SD) age of all the participants was 32±9 years and the mean BMI was 30±3 kg/m^2^.

### Design of included studies

The five included studies were randomized trials testing either different types of meals or medication in the respiration chamber. The participants were standardized with regard to exercise and alcohol intake from 10.00 pm the night before the EE measurements. The dinner meal was served at 18:00–19:15 after 3–6 h of fasting. The macronutrient compositions of the dinner meals are shown in Table 2. The dinner meal was followed by a postprandial period of 180 min with appetite and EE measurements (EE measurements from (15) are unpublished). The protocols for the stays in the respiration chamber in the included studies were not substantially different from each other. Physical activity was restricted in the hours prior to the measurements. All participants were sedentary for at least 100 min prior to and during the 180-min postprandial EE measurements. The only important difference was the ingestion of caffeine (in the form of one cup of tea or coffee) after the dinner meal during the EE measurement (40% of the included studies) (13–17). The Municipal Ethical Committee of Copenhagen and Frederiksberg, Denmark approved all included studies.

**Table 2:** Macronutrient composition in the dinner meals served in the respiration chambers

Reference	*n*	Carbohydrate E (%)	Protein E (%)	Fat E (%)
Raben et al. (15)	19	50	13	37
Hansen et al. (13)	32	48	15	37
Mikkelsen et al. (17)^1^	4	42	29	29
	4	43	28	29
	4	61	11	28
Larsen et al. (14)	10	52	13	35
Rasmussen et al. (16)^2^	19	45	15	40
	19	60	15	25

1 Total *n*=12 participants.

2 Total *n*=38 participants.

### Appetite measures and TEF

#### Satiety and TEF_iAUC_

**Fig. 2:**
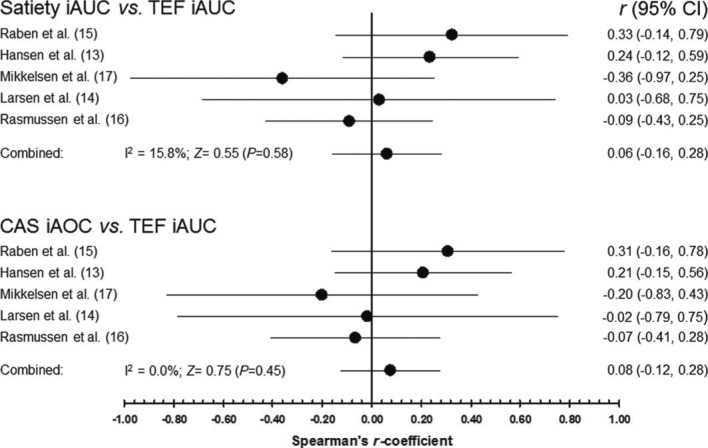
Forest plot of Spearman's *r*-coefficients. Every dot represents the individual study's *r*-value with 95% CI indicated by horizontal lines. The overall estimate (pooled random-effects model) from the meta-analysis and its CI are shown at the bottom of each subplot.

As illustrated in Fig. 2, the IPD meta-analysis found no overall association between satiety and TEF_iAUC_, with the Spearman's *r*-coefficients varying from −0.36 to +0.33, all being statistically non-significant (*P*>0.18). Pooling the data from these five individual trials produced a combined Spearman's *r*-coefficient of 0.06 (95% CI: −0.16 to 0.28), providing no support to the hypothesis of an association between satiety and TEF (*P*=0.58). This result was based on a small amount of heterogeneity, with a negligible degree of inconsistency (*I* ^2^= 15.8%). For sensitivity, we compare this with the more naïve approach – combining all participant data into one correlation analysis (i.e. ignoring the study structure), also resulting in an overall statistically non-significant Spearman's *r*-coefficient (*r*=0.079, *P*=0.42, *N*=109 observations). Inclusion of covariates did not affect the association between satiety and TEF_iAUC_ (see Supplementary file).

#### CAS and TEF_iAUC_

There was also no association between CAS and TEF_iAUC_ (*P*=0.45); the pooled Spearman's *r*-coefficient from these five trials was *r*=0.08 (95% CI: −0.12 to 0.28). As presented in Fig. 2, the *r*-values ranged from −0.20 to +0.31, all being statistically non-significant (*P*>0.20), and apparently with no inconsistency (*I* ^2^=0%). For sensitivity, combining all participant data into one correlation analysis also resulted in an overall statistically non-significant Spearman's *r*-coefficient (*r*=0.051, *P*=0.60, *N*=107 observations). Inclusion of covariates did not affect the association between CAS and TEF_iAUC_ (see Supplementary file).

### Associations between TEF_iAUC_, satiety, CAS, and EI from protein

**Fig. 3:**
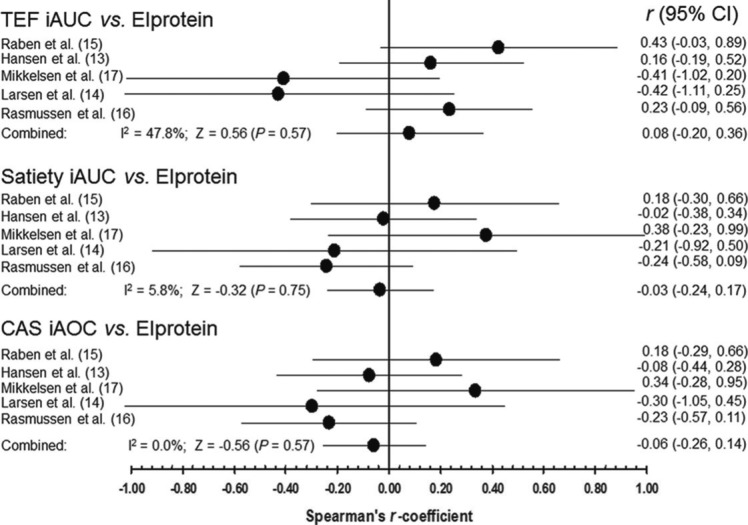
Forest plot of Spearman's *r*-coefficients. Every dot represents the individual study's *r*-value with 95% CI indicated by horizontal lines. The overall estimate (pooled random-effects model) from the meta-analysis and its CI are shown at the bottom of each.

Fig. 3 illustrates the three different associations with the protein intake in each study; upper part being in relation to TEF_iAUC_; middle being in relation to satiety; lower in relation to CAS.

No association between TEF_iAUC_ and protein intake was found (*P*=0.57), with the pooled Spearman's *r*-coefficient being 0.08 (95% CI: −0.20 to 0.36); with a moderate inconsistency across studies (*I* ^2^=47.8%). Combining all participant data into one correlation analysis (in analogy to a fixed-effects model) also resulted in an overall statistically non-significant Spearman's *r*-coefficient (*r*=0.08, *P*= 0.39, *N*=111 observations). However, we note that one study (15) showed a tendency toward a statistically significant association (*r*=0.43, *P*=0.068, *N*=19 observations). An association between TEF_iAUC_ and protein intake was observed (*P*=0.009) when including the covariates: Sex (*P*=0.01), caffeine intake (*P*<0.05), and EI in the dinner meal (*P*=0.02) (see Supplementary file).

Relating the satiety with protein intake did not indicate any association (*P*=0.75), as the pooled analysis showed a combined Spearman's *r*-coefficient of −0.03 (95% CI: −0.24 to 0.17); with a low degree of inconsistency (*I* ^2^=5.8%). The individual study results ranged from −0.24 to +0.38 with no indication of statistically significant results (*P*>0.15). Inclusion of covariates did not affect the association between satiety and protein intake (see Supplementary file).

Finally, for CAS in relation to the protein intake, the Spearman's *r*-coefficients varied from −0.30 to +0.34, all being statistically non-significant (*P*>0.18). Pooling the data from these five individual trials produced a combined Spearman's *r*-coefficient of −0.06 (95% CI: −0.26 to 0.14), providing no support to the hypothesis of an association between CAS and TEF (*P*=0.57). Inclusion of covariates did not affect the association between CAS and protein intake (see Supplementary file).

### Secondary TEF measures and appetite measures

Posthoc correlation analysis between TEF%_EI_ and satiety with all participant data combined resulted in an overall non-significant Spearman's *r*-coefficient (*r*=0.066, *P*= 0.49, *N*=109 observations). Similarly, correlation analysis between TEF% and CAS also produced a non-significant Spearman's *r*-coefficient (*r*=0.054, *P*= 0.58, *N*=107 observations).

Posthoc correlation analysis between TEF% and satiety including participant data from all included studies resulted in a non-significant Spearman's *r*-coefficient (*r*=0.134, *P*= 0.17, *N*=109 observations). Finally, similar analysis between TEF% and CAS showed a non-significant Spearman's *r*-coefficient (*r*=0.112, *P*= 0.25, *N*=107 observations).

## Discussion

We found no association between TEF_iAUC_ and the appetite measures, satiety, and CAS. Similarly, no associations were seen between TEF%_EI_ or TEF% and the appetite measures.

Previously, four studies examined the association between sensations of appetite and changes in EE following a meal (6, 46–48). Westerterp-Plantenga et al. (6) investigated this issue and found significant correlations between the differences (due to different macronutrient compositions in the test-meals) in both satiety and hunger over a 24-h period, and differences in TEF (expressed as 24-h TEF in kJ/day and TEF%_EI_). Another study by Westerterp-Plantenga et al. (48) observed a positive correlation between satiety and TEF%_EI_, but no association was found between hunger and TEF%_EI_. Crovetti et al. (46) found a positive correlation between fullness and TEF (expressed as AUC), but not between satiety and the desire to eat and TEF. Raben et al. (47) documented a positive correlation between satiety and TEF%_EI_, and between fullness and TEF%_EI_. However, no correlation was found between VAS measurements of hunger and prospective food intake (47). Overall, fewer than half of the appetite measures in these studies were correlated with TEF and thus the results are inconclusive.

Our results disclosed no association between TEF and satiety or CAS no matter which calculation method was used for TEF. This could be due to the fact that no association between protein and satiety was found. The lack of association between protein and satiety is supported by Raben et al., who found no difference in satiety when meals similar in energy densities but high in protein (31.8% of energy), fat, carbohydrate, and alcohol were ingested (49). Nevertheless, a recent review by Veldhorst et al. (7) concluded that the acute satiating effect of protein in single meals is present with a content of 25–81% of energy from protein. The mean (±SD) protein intake in our study was 15.6% (± 3.8) of energy from protein, so this could be too low to trigger a satiating effect. Due to the previously reported effect of protein on EE, we adjusted for the protein content in the statistical analysis (50, 51). Only 8 of the 111 participants in the present study received meals containing >20% of energy from protein and therefore no subgroup analysis was conducted on groups differing in protein intake. The results of this study may thus only be applicable to participants ingesting meals with normal protein contents (∼15% of energy from protein). In other words, the (lack of) associations between satiety and TEF may depend on the amount of protein ingested.

In the primary analyses of TEF and protein, the test for overall effect showed no association (*P*=0.57). However, including potentially confounding factors in the model resulted in an overall statistically significant Spearman's *r*-coefficient (*P*=0.009). This association between protein and TEF is supported by existing evidence. The reported TEF for protein is 20–30% of energy content compared to fat (0–3%) and carbohydrates (5–10%) (3). Thus, the higher the protein content of the meal, the larger the effect on EE (50, 51).

The participants in the present study had an average BMI of 30 kg/m^2^. One hundred of the 111 included subjects were in weight-stable conditions with study specific mean weight changes of no more than −0.2 kg±1.2 kg to 1.2 kg±3.9 over 28 days to 6 months. Only 11 of the included subjects had a weight loss of no more than 13.1±0.6 kg over 8 weeks prior to the measurements in the respiration chambers (13−17, 52). Numerous studies have reported a lower TEF in obese participants (53–55). However, other studies have reported no difference in TEF in lean and obese participants (56–58). A posthoc *t*-test analysis comparing the lower and upper quartiles for TEF showed a significant higher body weight in the upper quartile for TEF (*P*=0.02) but no difference between the quartiles was found for body fat (*P*=0.18). Whether obesity affects the association between satiety and TEF is also uncertain. This could possibly complicate the comparison of the present study with studies performed on normal-weight participants with a BMI < 25 kg/m^2^.

TEF has been reported to last longer than the 3-h measuring period used in this study and this could be a limitation. However, studies report that the major part of TEF takes place during the first few hours after ingestion of a meal (59, 60). Although these studies used meals containing less energy than those in our study, we propose that the 3-h period in our study still constitutes a valid measure of TEF. One study has suggested a measuring period of 6-h to increase the precision of the TEF measurement, but the highly non-significant results (*P*>0.48) in the present study makes it unlikely that a longer measurement period would alter these findings significantly (59).

TEF was measured in respiration chambers that contain large volumes of air which has to be exchanged continuously to produce valid measures of EE and small differences in TEF may therefore be difficult to detect in respiration chambers (61). This could limit the precision of the short-term (3-h) measurements of EE used in this study. However, based on experience from numerous respiration chamber studies at our department, we know that the chambers have a short response time: about 15 min for measuring larger differences in EE (bicycling) and about 60 min for detecting smaller differences in EE. Furthermore, the participants are not stressed in the chambers, in contrast to hood and mask measurement systems that may in particular affect their measured R-value. We therefore consider the respiration chambers to be a reliable and useful method for the purposes of this IPD meta-analysis.

All five included studies were conducted at our department and the respiration chambers and the study protocols were relatively similar, which implies a small variation between the studies. Furthermore, all protocols stated that the participants were sedentary during the 180-min postprandial EE measurement, meaning that physical activity during the TEF measuring period did not influence the results. However, this means that the results of these studies may all be affected by the same methodological issues. Similarly, it also increases the risk of our inclusion criteria being biased (62). Nevertheless, the present IPD meta-analysis is the first collection of a large sample of data on appetite and TEF in participants ingesting a meal containing approximately 15% of energy from protein.

If, in contrast to what our meta-analysis suggests, there actually exists an association between satiety and TEF, the question is whether this association is causal, or just the result of a protein induced concomitant elevation in both satiety and TEF, that is, temporal co-variation. The associations between protein and satiety (7, 46, 63, 64) and between protein and TEF (63–65), respectively, are both well documented. Furthermore, the higher the protein content, the higher both satiety and TEF will be. Nevertheless, the association between satiety and TEF, or the mechanisms potentially responsible for this possible association, is currently not well documented. Westerterp-Plantenga et al. (6) suggest that the increased oxygen consumption (and body temperature) associated with TEF may result in decreased oxygen availability, which may then induce satiety through unknown mechanisms. The authors based this suggestion on studies showing that high altitude, exercise, and chronic obstructive pulmonary disease, which are all characterized by limited oxygen availability, are all associated with higher satiety scores. However, in our study it is an unlikely explanation since the magnitude of TEF rarely reaches levels of oxygen shortage. The design of the present study does not allow for the investigation of causal associations and therefore, this requires further research in the future. Future studies should also consider the ranges of protein intake, the length of the EE measurement session, the number and frequency of appetite measures, and possible measurements of satiety-related hormones.

## Conclusion

In conclusion, the IPD meta-analysis found no association between satiety or CAS and TEF at protein intakes ∼15 E% of the meal (range 11–30 E%). The calculation method for TEF did not influence these findings. However, even though our study did not show any associations, this does not rule out the possibility that associations may be present at higher protein intakes.
